# Current and Emerging Approaches for Hepatic Fibrosis Treatment

**DOI:** 10.1155/2021/6612892

**Published:** 2021-07-17

**Authors:** Jingguo Li, Biguang Tuo

**Affiliations:** Department of Gastroenterology, Affiliated Hospital of Zunyi Medical University, Zunyi, China

## Abstract

Liver fibrosis resulting from chronic liver injury is a key factor to develop liver cirrhosis and risk of hepatocellular carcinoma (HCC) which are major health burden worldwide. Therefore, it is necessary for antifibrotic therapies to prevent chronic liver disease progression and HCC development. There has been tremendous progress in understanding the mechanisms of liver fibrosis in the last decade, which has created new opportunities for the treatment of this condition. In this review, we aim to make an overview on information of different potential therapies (drug treatment, cell therapy, and liver transplantation) for the liver fibrosis and hope to provide the therapeutic options available for the treatment of liver fibrosis and discuss novel approaches.

## 1. Introduction

Liver fibrosis is characterized as a reversible wound-healing process, which is mainly triggered by chronic liver tissue injury, including hepatitis B or C virus infection, alcohol, and nonalcoholic fatty liver disease (NAFLD) as well as autoimmune and genetic diseases [1]. Actually, liver fibrosis is a beneficial process at the beginning, because this process helps liver to repair its incomplete tissues and to restore its complete mass after the liver suffers from different kinds of acute injuries. However, if the injury is continuous and longstanding, liver fibrosis will unavoidably result in liver cirrhosis and even progress to hepatocellular carcinoma (HCC) eventually. During liver fibrosis, extracellular matrix (ECM), collagen I, and collagen III accumulate excessively and lead to scar formation due to the imbalance of fibrogenesis and fibrinolysis. Many studies focus on the cellular and molecular mechanisms of liver fibrosis to find effective treatment methods in clinic. With the deepening and broadening progressively of understanding for cellular and molecular mechanisms of liver fibrosis, more and more studies have been focusing on potent and effective drugs and novel therapies which target specific mechanism for patients with liver fibrosis or chronic liver diseases clinically. However, there are no any drugs or therapies showing significant benefit to patients on preventing or reversing hepatic fibrosis [2, 3]. In following sections, we will review recent advances of therapies of liver fibrosis, of which most are tested clinically now for NAFLD or nonalcoholic steatohepatitis (NASH) due to the high prevalence and dramatic significance in various chronic liver diseases leading to hepatic fibrosis.

## 2. Drug Therapy

Generally, the drug therapy is a traditional way to treat patients with liver fibrosis. As tremendous progress of drug therapy has been made in recent decades, people find great possibility and potential in drug therapy for liver fibrosis. We will review current potential antifibrotic drugs and further summarize them in Table 1. The drugs to treat liver fibrosis can be sorted out according to different targets and mechanisms.

### 2.1. Target to Oxidants, Apoptosis, and Inflammation

It is accepted widely that oxidative stress plays a key role in the progression of NASH [4, 5]. Consequently, people regard vitamin E as the treatment of NASH due to its well-known identity as a free radical scavenger. Previous studies showed that the administration of vitamin E for 1 year decreased serum transaminase activities and transforming growth factor-*β*1 in adult patients with NASH [6, 7]. Also, Sato et al. reported that vitamin E dramatically ameliorated hepatic steatosis, hepatocellular ballooning, inflammation, and decreased levels of serum hepatobiliary enzymes compared with the control group [8]. Furthermore, a clinical trial by Sumida and his colleagues showed that long-term administration of vitamin E over 2 years moderated liver fibrosis in NASH patients, and significant therapy effect was observed in patients with insulin resistance and serum transaminase activities [9]. Therefore, vitamin E is now considered as an optional treatment for NASH patients, even though the efficacy or safety of a longer-term administration should be confirmed and the potential toxicity of vitamin E for a long-term or high-dose administration, including increased all-cause mortality, hemorrhagic stroke, and prostatic cancer, should be taken into consideration as well [10–12]. Recently, Sebastiani et al. clarified that vitamin E improved hepatic steatosis in human immunodeficiency virus (HIV) monoinfected patients with a noninvasive diagnosis of NASH [13], and Klaebel et al. demonstrated that vitamin E and atorvastatin treatment, combined with a diet change, significantly improved NASH in a preclinical guinea pig model [14]. However, in a randomized controlled clinical trial, Bril et al. found that vitamin E therapy for 18 months did not show dramatic treatment effect in NASH patients with type 2 diabetes mellitus (T2DM) [15]. Currently, several further clinical trials concerning the efficacy of Vitamin E for NASH patients are ongoing (NCT02962297, NCT04193982, and NCT03669133).

GKT137831, which is a dual inhibitor of NADPH oxidase (NOX) 1/4, can inhibit both NOX 1 and NOX 4 to reduce reactive oxidative species (ROS) effect in HSCs. Aoyama et al. reported that GKT137831 showed significant improvement of liver fibrosis in mice induced by carbon tetrachloride (CCL4) and duct ligation [16]. What is more, in an ongoing clinical trial to assess safety and efficacy of GKT137831 in patients with primary biliary cholangitis, GKT137831 significantly improved serological cholestasis parameters and other relevant results (such as serum enzymes levels and liver fibrosis) will be released to the public soon later (NCT03226067).

The apoptosis of hepatic stellate cells (HSCs) is partly involved in the process of liver fibrosis regression; therefore, HSC apoptosis can be another potential target for liver fibrosis treatment. Apoptosis signal-regulating kinase 1 (ASK1) is activated by intracellular oxidative or endoplasmic reticulum (ER) stress to regulate some key pathways of cell death and inflammatory signaling via p38/c-jun N-terminal kinase (JNK) and mitogen-activated protein kinase (MAPK) in HSCs, macrophages, and hepatocytes [17, 18]. Accordingly, inhibition of ASK1 is recognized as a target for the therapy of liver fibrosis. Selonsertib (GS-4997), an inhibitor of ASK1, is being evaluated in combination therapy and monotherapy in clinical trials. A clinical trial on 67 NASH patients with moderate-to-severe fibrosis (stages 2-3) showed a clear regression in liver fibrosis, and the treatment effect is parallely related to other parameters of liver injury (improvements in progression to cirrhosis, fibrosis stage, liver fat content, and liver stiffness) in patients treated with selonsertib for 24 weeks [19]. Also, a clinical trial suggested that selonsertib prominently improved liver fibrosis and nonalcoholic fatty liver disease activity score (NAS) in NASH patients [20]. Recently, however, two phase III trials of selonsertib showed no significant antifibrotic effect in patients with NASH and bridging fibrosis F3/F4 with monotherapy of selonsertib for 48 weeks [21].

Emricasan, an irreversible oral pan-caspase inhibitor, ameliorates steatohepatitis and fibrosis through reducing excessive apoptosis and inflammation in murine NASH models [22] and decreases portal pressure with/without increased survival rate in rodent cirrhosis models [23, 24]. However, in a randomized placebo-controlled trial, emricasan showed no association with an significant improvement in hepatic venous pressure gradient (HVPG) or the occurrence of decompensation in patients with NASH cirrhosis (mostly compensated) and portal hypertension seemed to have small positive effect in the reduction of HVPG in compensated patients, especially who are with higher baseline HVPG [25]. Also in another trial, emricasan showed no or even less improvement in liver histology after 72 weeks treatment compared to placebo group in patients with NASH and F1-F3 fibrosis [26]. There are obvious difference in efficacy of emricasan for liver fibrosis between animal models, and human and a clinical trial about efficacy of emricasan treatment for patients with decompensated NASH cirrhosis is ongoing (NCT03205345).

### 2.2. Target to Hepatic Metabolism

NAFLD/NASH is associated with aberrant metabolism; therefore, numerous novel pharmacological therapies target aberrant metabolism, including free fatty acid production and lipotoxicity, insulin resistance and following lipolysis, extreme accumulation of triglyceride in hepatocytes and following disorder in autophagy and mitochondrial functions, excessive free fatty acids, and oxidative and endoplasmic reticulum stress [27]. Bile acid receptors, like Farnesoid X receptor (FXR), are a critical molecule of interacting some metabolic stress responses. Binding FXR with its ligand has the central function in reducing liver lipogenesis, glucogenesis, and steatosis [28]. 6-Ethylchenodeoxycholic acid, a semisynthetic bile acid derivative obeticholic acid (OCA), is a powerful agonist of FXR that can decrease liver fat and fibrosis and improve hepatic steatosis and portal hypertension in animal models [29–31]. A clinical trial of phases 2 and 3 including NASH patients, with and without T2DM, showed that OCA treatment for 6 weeks in patients with NASH and T2DM ameliorated insulin sensitivity as well as some parameters of fibrosis and inflammation in a dose-dependent way [32]. A double-blind, randomized, multicenter, placebo-controlled, phase 2 study with OCA including 283 patients with NASH, with and without T2DM, showed histological improvement on NASH and fibrosis in 110 patients of OCA treatment group compared to 109 patients of placebo group. Additionally, after 72 weeks therapy, 35% of patients receiving OCA compared to 19% receiving placebo showed a ≥1 stage improvement in liver fibrosis. However, more frequent pruritus (23% vs. 6%) and more often adverse changes with increases of low-density lipoprotein (LDL) and reduction of high-density lipoprotein (HDL) still existed. Besides, reduction of inflammatory and fibrotic secreted factors after OCA treatment was observed in vitro human liver system for NASH. In this study, an increase of apolipoprotein B (Apo B) secretion means abnormal lipoprotein metabolism [33], which is corresponding to the previous study about lipid profiles after OCA treatment [34]. Ratziu et al. conducted a clinical phase 3 trial which further assessed the efficacy of OCA treatment on liver histological features and development to cirrhosis, liver-related clinical outcomes, and mortality in patients with NASH-induced fibrosis [35], and an interim analysis of this phase 3 study suggested that OCA significantly improved critical components of NASH disease activity which is quite possible to predict clinical benefit [36]. Currently, another clinical trial with the purpose of defining the role of OCA in patients of NAFLD is ongoing (NCT03836937). Noticeably, FXR agonists without chemical similarities to bile acids have been being developed, which possess more beneficial effect and better tolerability on blood lipids [37].

Statins, a kind of lipid-lowering agent, is widely used clinically to reduce serum cholesterol levels via inhibiting 3-hydroxy-3-methylglutaryl coenzyme a reductase [38]. People have increasingly realized the potential of statins to treat liver diseases based on its properties. Previous studies reported that statins ameliorated fibrogenesis, liver inflammation, and oxidative stress in animal models of liver fibrosis [39–41]. Moreover, several retrospective analyses and clinical trials showed that statins reduced HVPG and risk of disease progression, including decompensation and HCC development even death, in patients with chronic liver diseases [42–45]. Currently, some clinical trials concerning statins treatment to liver cirrhosis are ongoing (NCT03780673, NCT02968810, and NCT04072601).

Another promising treatment targeting metabolism for NAFLD/NASH is the peroxisome proliferator-activated receptor (PPAR) family [46]. PPARs have three isotypes (PPAR*α*, PPAR*β*/*δ*, and PPAR*γ*) with different tissue distributions and ligand specificities. A widely various of ligands which bind these receptors can be synthesized. Pioglitazone, one of agonists of PPAR*γ*, has been widely examined for NAFLD [47]. Two randomized, double-blind, placebo-controlled studies showed that pioglitazone considerably improved necroinflammation and steatosis when compared to placebo in NASH patients with diabetes [48, 49]. Further, Cusi et al. verified the safety and efficacy of pioglitazone treatment after a long-term pioglitazone administration in 101 NASH patients with prediabetes/T2DM [50]. In addition to the histological improvement of NASH, Tavakoli et al. recently also reported that pioglitazone improved oxidative stress parameters (such as lipid peroxidation and total antioxidant capacity) in patients with NAFLD [51]. However, pioglitazone still has some potential risks and limitations in widely clinical routine, for example, gain of body weight and increase of heart failures, pancreas, and prostate cancer [52, 53]. Therefore, people have increasingly focused on combination of pioglitazone with other drugs for the therapy of NASH/NAFLD to reduce potential risks and limitations of pioglitazone, and currently, some relevant clinical trials are ongoing (NCT03950505 and NCT03646292).

Elafibranor (ELA), a dual agonist of PPAR*α*/*δ*, has been shown to ameliorate inflammation, steatosis, and fibrosis in animal models of NAFLD/NASH. Ratziu et al. reported the beneficial effect of elafibranor on resolving NASH without the deterioration of hepatic fibrosis after administration of 120 mg elafibranor in patients with NAS ≥ 4 [54], and another clinical trial has been evaluating the efficacy and safety of elafibranor in NASH without cirrhosis [55]. Noticeably, there is association between NASH patients with treatment of elafibranor and favorable changes in glucose profiles, lipids, inflammatory markers, and liver enzymes [54]. However, elafibranor has shown nothing significant effect on liver fibrosis, which leads the way of future therapy for NASH to combination of drugs personally [56]. Roth et al. reported that additive metabolic and histological effects were improved by the administration of combining OCA with ELA in amylin liver NASH (AMLN) diet *ob*/*ob* mice [57]. Also, an announcement (from Genfit) demonstrates that elafibranor has shown dramatic treatment effect in phase 2 study in patients of primary biliary cholangitis (PBC) with favorable tolerability and less safety concerns (NCT03124108). Currently, a phase 3 study to evaluate the efficacy and safety of elafibranor in patients with NASH is ongoing (NCT02704403).

Saroglitazar, a dual agonist of PPAR*α*/*γ*, has already been approved to ameliorate dyslipidemia in patients with diabetes in India [58]. Saroglitazar showed a dramatic improvement on liver histopathology and biochemistry in NASH mouse models [59]. Hassan et al. demonstrated that saroglitazar successfully improved NASH via inhibiting the pathway of hepatic lipopolysaccharide (LPS)/toll-like receptor 4 (TLR4) and the dysfunction of adipocyte in rats with high-fat emulsion/LPS model-induced NASH [60]. Interestingly, Makled and his coworkers recently reported another pathway of saroglitazar affecting liver fibrosis, which means that saroglitazar is able to improve liver fibrosis in TAA-induced liver fibrosis models of rats via suppressing leptin, transforming growth factor-*β*1 (TGF*β*1), tissue inhibitor of metalloproteinases-1 (TIMP-1), and platelet-derived growth factor-BB (PDFG-BB) [61]. Currently, several further clinical trials to test treatment effect of saroglitazar for NAFLD/NASH (NCT03863574 and NCT03061721) are under conducting.

### 2.3. Target to Gut Microbiome

“Gut-liver axis” is an anatomical and functional unit comprised by the intestine and the liver. There are many interactions between the gut and the liver via the portal vein and the bile [62]. Patients with liver fibrosis and even cirrhosis have structural disorders in the gut-liver axis, especially changes in the composition and diversity [62–65]. Therefore, a novel and exciting proposal on modulating gut microbiome as an antifibrotic therapy is proposed.

IMM124-e is extracted from bovine colostrum of cows immunized against LPS. IMM124-e is able to diminish exposure of the liver to bacterial products derived from gut and LPS. A clinical trial showed that IMM124-e improved liver enzymes and glycemic regulation by increasing serum glucagon-like peptide 1 (GLP-1), adiponectin, and T regulatory cells [66]. The efficacy and safety of IMM124-e in patients with NASH and severe alcoholic hepatitis have been tested in clinical trials (NCT02316717 and NCT01968382).

Solithromycin, a strongly effective macrolide antibiotic, has shown in a phase 2 open-label trial that NAS (average reduction, 1.3) and alanine aminotransferase (ALT) serum level (average reduction, 17.8 U/L) was diminished after treatment with solithromycin for 90 days (NCT02510599).

Another possible way to target gut-liver axis is to simulate “favorable signals” stimulated by gut-derived hormones. One of these hormones is fibroblast growth factor (FGF) 19 in human (FGF15 in the mouse). Besides the regulation of bile acid synthesis, FGF19 is involved in several favorable metabolic signal pathways in liver cells [27]. NGM282, an engineered analogue of FGF19, dramatically decreased the content of liver fat after 12 weeks treatment in a clinical trial of phase 2 with 82 NASH patients with stage 1-3 liver fibrosis [67]. Additionally, another single-center study involving 19 patients demonstrated a reduction of histological fibrosis in 42% of candidates after 12-week therapy of NGM282 [68]. Currently, a randomized clinical trial to evaluate treatment effect of NGM282 is under conducting with 152 patients of NASH and stage 2/3 fibrosis (NCT03912532).

Fecal microbiota transplantation (FMT) is also a novel and promising way to affect gut microbiome to work on liver. FMT is to transfer some fecal content from healthy people to the intestine of patients. Ferrere et al. reported that FMT improved liver injury in mouse models induced by alcohol [69]. What is more, in CCL4-induced rat models, Wang et al. proved that FMT was better than probiotics to prevent hepatic encephalopathy, which attributes to the protection on intestinal mucosal barrier function [70]. In rather few clinical trials of FMT, it is noticeable that FMT reduced hepatic inflammation in patients with severe alcoholic hepatitis (SAH) during 1-year follow-up [71]. Particularly, another clinical trial showed that FMT improved cognition and decreased hospitalizations in 20 patients with cirrhosis and recurrent hepatic encephalopathy when compared to normal care [72].

Targeting to gut microbiota for curing liver fibrosis still has a long way to go. For example, it is not exactly possible for some “broad” nonselective interventions like antibiotics, probiotics, and even fecal microbiota to treat hepatic fibrosis [73]. However, if personal treatment of microbiota can be applied, which is similar to the treatment of liver encephalopathy by transplantation of fecal microbiota to cirrhotic patients, things may be different.

### 2.4. Target to Hepatic Fibrosis

Various targets discussed above are indirect ways to treat liver fibrosis; however, what is important, that acts as a predictor to mortality and survival time to development of severe liver disease in biopsy-proven NAFLD, is fibrosis stage but not NASH. There is also an urgent need for effective antifibrotic therapy to cure patients with final-stage fibrosis in a direct way.

The C-C chemokine receptor (CCR) 5 is an important contributor in fibrosis progression and HSC-Kuppfer cell interaction, which makes it possible for cenicriviroc, an antagonist of CCR2/CCR5, to be a potential antifibrotic agent. The inhibitor of CCR5 is predicted to inhibit HSCs from activation, proliferation, and migration [74]. Lefebvre and coworkers found that cenicriviroc has dramatic positive effects on anti-inflammation and antifibrosis in animal fibrosis models [75]. Furthermore, Friedman et al. demonstrated the antifibrotic effect of cenicriviroc in a phase 2 clinical trial and in a phase 2b trial containing 289 NASH patients with significantly improved liver fibrosis but no worsening steatohepatitis (20%) when compared with placebo group (10%) after 1 year of cenicriviroc therapy [76]. These results lay cement foundation to the phase 2b/3 trials of cenicriviroc safety and efficacy which are currently ongoing (NCT03517540) [77, 78].

Galectin-3 protein plays a key role in regulating myofibroblast activation and hepatic fibrosis [79]. GR-MD-02 (also called belapectin), an inhibitor of galectin-3, displayed a significant improvement in hepatic histological feature with marked reduction in collagen deposition and NASH activity [80]. In a randomized clinical study, GR-MD-02 was safe for administration in patients with NASH and stage 3 fibrosis, while it showed nothing obvious therapy effect in three noninvasive imaging modality tests for evaluation of liver fibrosis [81, 82]. A phase 2b clinical trial, involving 162 patients with NASH, cirrhosis, and portal hypertension, showed that GR-MD-02 had no association with marked amelioration in fibrosis or HVPG as compared with placebo after 1 year of therapy. However, 2 mg/kg belapectin indeed reduced HVPG in a subgroup analysis of patients without esophageal varices [83]. Currently, a study evaluating the efficacy and safety of belapectin for the prevention of esophageal varices in NASH cirrhosis is ongoing (NCT04365868).

PRI-724 (alias ICG-001) is an inhibitor of CBP/*β*-catenin, which has shown antifibrotic effect through inhibiting expression of alpha-smooth muscle actin (*α*-SMA) and collagen 1. Previous studies reported that PRI-724 attenuated HSC activation and ECM accumulation in CCL4-induced mouse model of liver fibrosis and decreased fibrosis severity in liver fibrosis mouse model induced by hepatitis C virus (HCV) [84, 85]. Noticeably, a single-center, open-label phase I trial showed that after the administration of PRI-724 in patients with HCV cirrhosis, Child-Pugh score increased at 1-3 points in four patients in 40 mg/m2/day and 160 mg/m2/day group and histological improvement was found in 3/12 patients in 10 mg/m2/day and 40 mg/m2/day group while histological deterioration was found in 2/12 patients in 10 mg/m2/day [86]. Currently, a phase I/IIa clinical trial to assess safety and effectiveness of PRI-724 for patients with liver cirrhosis derived from hepatitis C or B virus is ongoing and estimated to be completed in November 2020 (NCT03620474).

Heat shock protein 47 (HSP47), a collagen 1 chaperone, plays a key role in collagen 1 synthesis. Sato et al. reported that Hsp47 siRNA containing vitamin A-coupled liposome had significant antifibrotic effect by reducing production of collagen 1 in 3 models of liver fibrosis [87]. BMS 986263 is HSP47 siRNA delivering lipid nanoparticle, which has already been tested for safety in healthy human, and in addition, a phase 1b/2 trial to evaluate safety and tolerability of BMS 986236 in patients with moderate to extensive liver fibrosis was completed in 2016 (NCT01858935 and NCT02227459).

## 3. Cell Therapy

Cell therapy, an emerging method for treating liver fibrosis, acts as an alternative to liver transplantation. Cell therapy is aimed at preventing progression of liver fibrosis by the engagement in regulation of cell activities in liver, remodeling of ECM, and stimulation of parenchyma in a paracrine way [88]. We will review current potential cell therapy and further summarize them in Table 2.

## 4. Endothelial Progenitor Cells

Endothelial progenitor cells (EPCs) are able to differentiate into mature endothelial cells with the purpose of angiogenesis and vasculogenesis and secretion of cytoprotective and nutritious factors in a paracrine way on tissue cells. These traits enable EPCs to be cellular therapeutic target in some strategies of regenerative medicine, such as tissue engineering approaches or cellular transplantation [89]. Beaudry et al. reported that the regeneration rate of liver tissue relied on the incorporation and mobilization of EPCs after partial hepatectomy, indicating the importance of EPCs in liver regeneration after hepatectomy [90]. Therefore, EPC-based therapy is investigated widely for restoring liver function in liver fibrosis. In different animal models, EPC transplantation indeed increased survival rate and improved liver fibrosis via suppressing HSCs, reducing serum levels of aspartate aminotransferase (AST) and ALT, and increasing hepatocyte proliferation and expression of hepatocyte growth factor (HGF) and vascular endothelial growth factor (VEGF) [91–95]. These results boosted clinical huge interest in EPC therapy for liver fibrosis. A phase 1/2 clinical study suggested that there was no serious side-effects observed in EPC therapy for liver cirrhosis after one-year follow-up; therefore, it is feasible and safe to conduct EPC transplantation in patients with advanced liver cirrhosis via liver artery. In addition, transplanted EPCs also ameliorated liver function and portal hypertension temporarily but significantly. As for several assessed parameters, D'Avola et al. summarized that quality of expanded EPCs in vitro was steady in cirrhosis stage and other expanded cells displayed phenotype similar to active EPCs with production of HGF, insulin like growth factor 1 (IGF-1), VEGF, and epidermal growth factor (EGF) [96]. A randomized, controlled phase 3 trial is currently ongoing (NCT03109236) and expected to elucidate the possible clinical benefits of advanced cirrhosis patients treated by EPC-based therapy.

## 5. Bone Marrow Mononuclear

Adult bone marrow is well-known as an origin of bone, blood, and vascular tissues. Bone marrow transplantation, one of the earliest established cell therapy technologies, has been broadly applied in the treatment of immunological deficiency disorders and leukemia for many years. In the last decade, however, studies suggested that bone marrow mononuclear cell (BMMN) transplantation poses positive effect on liver function and liver fibrosis. In 2010, Carvalho et al. found a reduction in the expression of laminin, collagen I and IV, and the amount of bile ducts after BMMN transplantation in rats with cholestatic fibrosis [97]. In 2013, they further clarified that BMMN transplantation upregulated the expression of matrix metallopeptidase- (MMP-) 9 and MMP-13 with the downregulation of TIMPs and promotion of fibrogenic cell apoptosis in the same animal models, which contribute to ECM degradation [98, 99]. Additionally, de Andrade et al. in 2015 reported that BMMN treatment improved mitochondrial bioenergetics via stimulation of liver oxidative capability, reduction of oxidative stress, regulation of mitochondrial coupling, and biogenesis in liver fibrosis [100]. Currently, a further clinical trial is still ongoing (NCT03468699).

## 6. Mesenchymal Stem Cells

Mesenchymal stem cells (MSCs) were firstly described in the 1980s and defined as cells in bone marrow, attaching to surfaces and featuring a spindle-shaped morphology [101]. At present, it is well-known that the MSC has therapeutic potential for liver disease due to its nonimmunogenicity, homing to injured sites, differentiation capacity, release of molecules, and immunomodulatory properties [102–104]. However, argument exists in antifibrotic effects of MSCs. Some papers indicated that bone marrow-derived MSCs not only fail to improve liver fibrosis [105, 106], but also have potential to become myofibroblasts and HSCs which can boost development of liver fibrosis [107, 108]. Oppositely, Zhao et al. reported that MSCs would show better antifibrotic effects on liver under injection at earlier times during liver injury [109]. Anyway, abovementioned data lacks conclusiveness, and further studies for MSC therapy in liver fibrosis are very necessary. Then, we will further demonstrate details about bone marrow mesenchymal stem cells (BMMSCs) and adipose-derived mesenchymal stem cells(ADSCs) which are the two most common sources of MSCs,

As the result of the earliest isolation and identification, BMMSCs were applied most in clinical study for therapy of various diseases, such as hepatic fibrosis [110]. Benefits of BMMSC therapy include collagen decrease, HSC apoptosis, proinflammatory cytokine reduction, and liver enzyme recovery. Recently, people found that BMMSCs with transfection of hepatocyte nuclear factor 4 alpha (HNF-4*α*) presented more benefits than untreated BMMSCs in a CCL4-induced animal model, which means that HNF-4*α*-BMMSC transplantation improved parameters of liver function and HNF-4*α* exerted positive effect on MSC anti-inflammation [111, 112]. It also has been demonstrated that BMMSCs promoted regeneration of liver by upregulating expression of HGF and MMP-2 and downregulating expression of cytokeratin-19 (CK-19) in a rat model of BDL-induced liver fibrosis [113]. However, some studies indicated that BMMSCs acted as contributor to maintain fibrosis and accounted for liver fibrosis progression [108, 114]. Currently, clinical phase 1/2/3 trials (NCT03838250, NCT00993941, and NCT01854125) concerning BMMSC transplantation and a randomized controlled study to confirm the long-term effect and safety of BMMSC treatment for liver cirrhosis patients (NCT03209986) are under conducting.

ADSCs have potential in therapy of regenerative medicine due to the convenience of being acquired from liposuction procedures and propagated ex vivo [115]. Hao et al. made comparison between ADSCs and BMMSCs indicating the efficacy of both cells' treatment in liver fibrosis, while ADSCs presented better promotion to inhibit activation, proliferation, and activation of HSCs and the level of AST/ALT, which lays foundation for ADSCs being a nice optional treatment for hepatic fibrosis [116]. Many studies proved the effectiveness of ADSCs in the treatment of liver fibrosis in animal models [117–122], and interestingly, Zhang et al. found that cultivating ADSCs in 3D environment presented higher expression of IGF-1, HGF, and interleukin-6 (IL-6) as compared with 2D environment, leading improvement of liver function after transplanting spheroids to a liver fibrosis mouse model [123]. Even though ADSCs are perceived as an assisted tool in regeneration medicine of liver and have considerable potential in treatment of liver injuries, argument on timing of transplantation, consequence of short and long-term use, and the route of administration still exist. Therefore, it is necessary for further studies to explore more specific and accurate mechanisms of liver regeneration. Currently, several clinical trials concerning ADSC transplantation are ongoing (NCT02705742, NCT04088058, NCT03629015, and NCT03254758).

## 7. Primary Hepatocytes

The quality of reproduction of liver cells has been recognized as a beneficial condition for amplification of cells in vitro; however, cultivating cells in 2D systems fails to provide essential signaling milieu for preserving primary liver cells out of the liver, which triggers the loss of normal morphology and physiological function of primary hepatocytes. Over decades, scientists succeed in maintaining human primary hepatocytes in simulated-physiological condition with 3D scaffolds and bioreactor systems [124]. With regard to the effectiveness of primary or lineage hepatocytes in liver fibrosis therapy, cell therapy in animal models generally showed more beneficial than that in human. Ito et al. found that in bile duct ligation-induced Nagase analbuminemic rats, the transplantation of primary liver cells improved albumin and bilirubin serum levels and survival [125]. Another study suggested that transplanting alginate-encapsulated hepatocytes with immortality had therapeutic effects (improving prothrombin time, survival, bilirubin, and serum albumin levels) in a model of hyperammonemia-induced hepatic encephalopathy, which is similar to the results of transplanting primary hepatocytes in the same model [126]. Despite huge progresses in creating ideal cultivation of nonautologous primary hepatocytes have been made, there are many challenges waited to be overcome, including immune rejection and viability loss after cryopreservation/thawing cycles [127].

## 8. Hepatic Progenitor Cells

Oval cells or hepatic progenitor cells are involved in duct reaction which is observed following certain hepatic injuries. Duct reaction is stimulated by intermediate ductular cells and forms new bile ducts which are associated with hepatic fibrosis [128]. Particularly, oval cells can transform into hepatocytes and/or duct cells when the proliferation of both two cell types is hindered, which qualifies oval cells for potential cell targets of liver fibrosis therapy [129]. Actually, Liu et al. reported that oval cells possibly led to liver fibrosis by mesenchymal transition after liver orthotopic transplantation in small-for-size fatty liver grafts [130], and besides, Lotowska et al. reported the proliferation and activation of duct cells, which associated with HSCs, macrophages, and portal cells, promoted liver fibrogenesis in a BDL model [131]. However, the engagement of oval cells with liver regeneration via providing new parenchymal cells was also confirmed by numerous studies. In 2017, Awan et al. reported that in CCL4-induced fibrosis models, oval cells derived from MSCs are beneficial to liver regeneration [132], and Addante et al. showed in 2018 that bone morphogenetic protein 9 (BMP9) was confirmed to inhibit reaction of duct and activation of oval cells to reduce liver regeneration in BMP9-deficient mice with DDC-induced cholestasis [133]. All results abovementioned embody two distinct roles of oval/hepatic progenitor cells in liver fibrosis and also emphasize the importance and necessity of further study for molecular mechanisms of oval/hepatic progenitor.

## 9. Pluripotent Stem Cells

Because of the complex morphophysiology, specialized functions, and intricate interactions with various hepatic parenchymal cells, it is really challenging to obtain functional hepatocytes from stem cells [134]. Over several years, despite so-called hepatocyte-like cells were generated from pluripotent cells, these cells which have most hepatocyte features generally lack some properties concerning the protein expression, gene expression profile, and/or metabolism [135]. To adapt future patient-specific protocols, Takahashi and Yamanaka indicated that induced pluripotent stem cells (IPSCs) generated from cells should be considered as a great candidate [136]. Despite some safety issue of the residual teratogenic/tumorigenic activity in differentiated cells exist [137], IPSCs still are worth being applied in liver fibrosis therapy when their safety and effectiveness are confirmed. There have been studies showing several successful cases of generating human hepatocyte-like cells from different types of PSCs [137, 138], and other recent preclinical trials also showed promising results of PSC therapy in different liver fibrosis models [139–141].

## 10. Liver Transplantation and Extension

Final-stage liver fibrosis with different complications is extremely dangerous to human life with high mortality and morbidity. Liver transplantation (LT) currently is the broadly accepted only lifesaving therapy option for patients with advanced liver fibrosis. The first successful case of liver transplantation with the development of managing immunosuppressant therapies, improved treatment of complications after surgery, and more proper matching of donor-recipient was reported, and the evaluations of short- and long-term of posttransplanted patients have improved increasingly [142]. Even though LT dramatically improves survival rate of patients with advanced fibrosis, we have to witness the broadening gap between the number of patients waiting for liver transplantations and the number of available and appropriate donors, which causes roughly 15% mortality rate of waiting patients in LT. In the future, the protocol of LT will be aimed at looking for the donor pool expansion approaches and available systems. Increasing the survival rate of liver transplanted patients will be more challenging, and proper clinical allocation of livers and ethical standards still play a key role in decisions.

Hepatic organoids are considered as functional 3D models in vitro with maintaining important physiological traits of the liver [143]. Generally, liver organoids can be acquired from expansion and isolation of hepatic progenitor cells or stem cells. Transplanting hepatic organoids into mouse liver failure models improved liver function partly, showing the capability for engrafting and repopulating injured liver [143]. Another study demonstrated that transplanting liver organoids into mice with acute liver injury could secret alpha-1-antitrypin and human albumin with similar levels to these mice receiving adult hepatocyte transplantation [144]. Coculture of endothelial and mesenchymal cells, combined with iPSCs, enabled 3D liver tissue to generate hepatocytes. These liver buds can produce serum proteins and perform detoxifying after they become mature and vessels [145]. Currently, relevant study is focusing on clinically applying liver buds proper to liver administration through the portal vein in patients waiting for liver transplantation [146].

Liver tissue engineering is also able to reduce the waiting patients via producing extracorporeal hepatic equipment and biocompatible scaffolds which apply well in vivo and/or in vitro [147]. In tissue engineering, a 3D physiological microenvironment is critical for developing tissue models in vitro [148]; therefore, it is very important to look for efficient biocompatible scaffolds with ideal materials, and currently, major approaches are based on biomaterials, including decellularized ECM, polymer-based 3D and bioprinting 3D constructs, bioreactors, and liver-on-chip [149]. Overall, these cutting-edge technologies are promising in the treatment of final-stage liver diseases. The current task is also to examine how to develop and apply them in clinical practice.

## 11. Conclusion

In conclusion, hepatic fibrosis is triggered by complex mechanisms which make it extremely difficult for choosing proper methods to treat patients with liver fibrosis. The explicit fact now is that before we get the key to unlock the mysterious world of liver fibrosis, liver transplantation is still the most direct and effective way to resolve it. Fortunately, however, people are getting consensus progressively that it is especially important to customizing personal treatment according to different scenarios in patients of liver fibrosis. Therefore, it can be predicted easily that combination therapy for liver fibrosis among different drugs, even between the drug and the cell therapy, will become predominant trend in future liver fibrosis therapy. Certainly, more clinical trials regarding therapy of liver fibrosis and experimental studies concerning mechanism of liver fibrosis are still needed currently.

## Figures and Tables

**Table 1:** Potential antifibrotic drugs for hepatic fibrosis.

Drug	Mechanism	Clinical phase
Vitamin E	Antioxidants	II/III
GKT137831	Antioxidants	II
Selonsertib (GS-4997)	ASK1 inhibitor	III
Emricasan	Caspase inhibitor	II
Obeticholic acid	FXR agonist	III
Statins	Lipid-lowering agents	II/III/IV
Pioglitazone	PPAR*γ* agonist	IV
Elafibranor	PPAR*α*/*δ* agonist	III
Saroglitazar	PPAR*α*/*γ* agonist	II
IMM124-e	LPS immune	II
Solithromycin	Macrolide antibiotic	II
NGM 282	FGF 19 analogue	IIb
Cenicriviroc	CCR2/CCR5 antagonist	II/III
GR-MD-02	Galectin-3 inhibitor	IIb
PRI-724	CBP/*β*-catenin inhibitor	I/IIa
BMS 986263	Anti-HSP47	Ib/II

**Table 2:** Potential cell therapy for hepatic fibrosis.

Cell therapy	Mechanism	Clinical phase
Endothelial progenitor cells	Angiogenesis and vasculogenesis and secretion of cytoprotective and nutritious factors in a paracrine way on tissue cells	III
Bone marrow mononuclear	An origin of bone, blood, and vascular tissues	I/II/III
Primary hepatocytes	High quality of reproduction	Unknown
Hepatic progenitor cells	Hepatic progenitor cells can transform into hepatocytes and/or duct cells when the proliferation of both cell types is hindered	Unknown
Pluripotent stem cells	Take possession of most hepatocyte features	Unknown
